# Low ^15^N Natural Abundance in Shoot Tissue of *Brachiaria humidicola* Is an Indicator of Reduced N Losses Due to Biological Nitrification Inhibition (BNI)

**DOI:** 10.3389/fmicb.2018.02383

**Published:** 2018-10-04

**Authors:** Hannes Karwat, Konrad Egenolf, Jonathan Nuñez, Idupulapati Rao, Frank Rasche, Jacobo Arango, Danilo Moreta, Ashly Arevalo, Georg Cadisch

**Affiliations:** ^1^Institute of Agricultural Sciences in the Tropics (Hans-Ruthenberg-Institute), University of Hohenheim, Stuttgart, Germany; ^2^International Center for Tropical Agriculture (CIAT), Cali, Colombia

**Keywords:** δ^15^N isotopic discrimination, ^15^N:^14^N fractionation, soil microbial nitrification, N uptake, N assimilation, nitrate leaching, soil incubation

## Abstract

The tropical forage grass *Brachiaria humidicola* (Bh) suppresses the activity of soil nitrifiers through biological nitrification inhibition (BNI). As a result, nitrate (${\text{NO}}_{3}^{-}$) formation and leaching are reduced which is also expected to tighten the soil nitrogen (N) cycle. However, the beneficial relationship between reduced ${\text{NO}}_{3}^{-}$ losses and enhanced N uptake due to BNI has not been experimentally demonstrated yet. Nitrification discriminates against the ^15^N isotope and leads to ^15^N depleted ${\text{NO}}_{3}^{-}$, but ^15^N enriched ${\text{NH}}_{4}^{+}$ in soils. Leaching of ^15^N depleted ${\text{NO}}_{3}^{-}$ enriches the residual N pool in the soil with ^15^N. We hypothesized that altered nitrification and ${\text{NO}}_{3}^{-}$ leaching due to diverging BNI magnitudes in contrasting Bh genotypes influence soil ^15^N natural abundance (δ^15^N), which in turn is reflected in distinct δ^15^N in Bh shoot biomass. Consequently, high BNI was expected to be reflected in low plant δ^15^N of Bh. It was our objective to investigate under controlled conditions the link between shoot value of δ^15^N in several Bh genotypes and leached ${\text{NO}}_{3}^{-}$ amounts and shoot N uptake. Additionally, plant ^15^N and N% was monitored among a wide range of Bh genotypes with contrasting BNI potentials in field plots for 3 years. We measured leaf δ^15^N of young leaves (regrown after cutback) of Bh and combined it with nitrification rates (NRs) of incubated soil to test whether there is a direct relationship between plant δ^15^N and BNI. Increased leached ${\text{NO}}_{3}^{-}$ was positively correlated with higher δ^15^N in Bh, whereas the correlation between shoot N uptake and shoot δ^15^N was inverse. Field cultivation of a wide range of Bh genotypes over 3 years decreased NRs in incubated soil, while shoot δ^15^N declined and shoot N% increased over time. Leaf δ^15^N of Bh genotypes correlated positively with NRs of incubated soil. It was concluded that decreasing plant δ^15^N of Bh genotypes over time reflects the long-term effect of BNI as linked to lower ${\text{NO}}_{3}^{-}$ formation and reduced ${\text{NO}}_{3}^{-}$ leaching. Accordingly, a low δ^15^N in Bh shoot tissue verified its potential as indicator of high BNI activity of Bh genotypes.

## Introduction

Biological nitrification inhibition (BNI) by the tropical forage grass *Brachiaria humidicola* (Sylvester-Bradley et al., 1988; Subbarao et al., 2007) is an ecologically evolved trait to compete with nitrifying soil organisms for available ammonium (${\text{NH}}_{4}^{+}$). Certain root derived exudates (Coskun et al., 2017a), e.g., brachialactone, have been verified to block the activity of ammonia monooxygenase (AMO) and hydroxylamine oxidoreductase (HAO) pathways in nitrifiers such as *Nitrosomonas europaea* (Subbarao et al., 2009). By preventing the microbial transformation of less soil mobile ${\text{NH}}_{4}^{+}$ to more soil mobile nitrate (${\text{NO}}_{3}^{-}$), BNI is expected to reduce leaching of nitrogen (N) from ecosystems (Subbarao et al., 2009, 2013; Coskun et al., 2017b). Recent research has been undertaken to investigate the BNI effect by Bh on N dynamics, for example on the reduction of nitrous oxide (N_2_O) emissions from soils (Byrnes et al., 2017), or on the influence of residual BNI effect on N uptake of subsequent crops (Karwat et al., 2017). However, indicators for reduced ${\text{NO}}_{3}^{-}$ leaching losses by effective BNI do not exist yet. The reduction of ${\text{NO}}_{3}^{-}$ leaching from agroecosystems due to BNI is one of the claimed central features in terms of BNI implementation (Subbarao et al., 2013; Coskun et al., 2017b).

Nitrification and N leaching are two main soil processes that lead to ^15^N:^14^N isotope fractionation (Mariotti et al., 1981). This results in a ^15^N enriched ${\text{NH}}_{4}^{+}$ and a ^15^N depleted ${\text{NO}}_{3}^{-}$ pool in soils (Delwiche and Steyn, 1970; Herman and Rundel, 1989; Jones and Dalal, 2017). Under high rainfall conditions, nitrification-derived ^15^N-depleted ${\text{NO}}_{3}^{-}$ is exposed to high leaching, whereby plants then would feed on remaining ^15^N enriched ${\text{NH}}_{4}^{+}$. In contrast, plants with effective BNI are expected to suppress microbial ${\text{NO}}_{3}^{-}$ formation. Consequently, less ^15^N depleted ${\text{NO}}_{3}^{-}$ would be lost from the system. Under both scenarios, Bh feeds mainly on ${\text{NH}}_{4}^{+}$, toward which it has a higher tolerance than other *Brachiaria* species (Castilla and Jackson, 1991; Rao et al., 1996). The ^15^N signature of soil mineral N can be reflected in a corresponding shoot ^15^N signature (Takebayashi et al., 2010). Previous field studies demonstrated that foliar δ^15^N increased in conjunction with increasing nitrification and N leaching by precipitation (Pardo et al., 2002; Stamatiadis et al., 2006; Huber et al., 2013; Yé et al., 2015). From these studies, the relationship between plant δ^15^N and soil nitrification and N losses was apparent, which indicates a possible strong relationship between BNI and the δ^15^N in plant tissue.

What remains unclear is if shoot δ^15^N of Bh genotypes is linked to plant induced BNI, and if this relates to reduced nitrification, thus reduced ${\text{NO}}_{3}^{-}$ leaching. We therefore hypothesized that (i) effective BNI is linked to reduced soil nitrification rates (NRs), enhanced N nutrition, reduced ${\text{NO}}_{3}^{-}$ leaching; and that (ii) this link is expressed in respective low δ^15^N in plant shoot tissue due to nutrition of Bh on a less naturally enriched N pool of ^15^N. In contrast, it was expected that, respectively, higher NRs and higher ${\text{NO}}_{3}^{-}$ leaching is expressed in higher δ^15^N of shoot biomass due to the uptake of ^15^N enriched soil N.

## Materials and Methods

### Experiment 1: Relationship Between δ^15^N of Bh, N Uptake, and ${\text{NO}}_{3}^{-}$ Leaching Under Controlled Conditions

A greenhouse study at the University of Hohenheim (UHOH), Stuttgart, Germany, was implemented as an α-design, i.e., a design with incomplete blocks, that are grouped into complete replicates. The trial contained four complete replicates with two blocks per replicate. The aim was to monitor the effect of N loss and N uptake after N fertilization on the δ^15^N in shoot biomass of different Bh genotypes. Experimental soil columns were manufactured from PVC-drainpipes (Ø 11 cm × 100 cm) in order to enable deep rooting of Bh and monitoring of ${\text{NO}}_{3}^{-}$ dislocation within the soil profile. A fossil tertiary clay loam (collected in Lich, Germany, 50°31′2.0”N, 8°50′55.9”E) with pH 5.7, 36% clay content, 0.25% total C, and 0.025% total N was used. The original soil was amended with sand (25 vol%) in order to improve drainage, and the resulting substrate was filled into the experimental pipes.

Five apomictic Bh hybrids (Bh08 selection: Bh08-1149, Bh08-700, Bh08-675, Bh08-696, Bh08-1253) and two germplasm accessions of Bh (CIAT 16888, CIAT 679 cv. Tully) were used as test genotypes (Rao et al., 2014). Bh stolons were first propagated from a Bh stock collection at UHOH and transferred to a turf-based culture substrate for 3 weeks for root establishment. Then, the young Bh plants were transplanted in August 2014 to the experimental pipes were exposed to supplementary artificial light (photosynthetically active radiation averaging 800 μmol m^−2^ s^−1^) for 12 h photoperiod. Day and night temperature regimes were adjusted to 25 and 20°C, respectively. Plants were raised with a basal fertilizer of N-P-K-S (analog to 30-60-150-35 kg ha^−1^). After the establishment phase of 6 weeks, plants were cut to 2–3 cm height to facilitate regrowth. The experimental phase commenced with application of 150 kg N-${\text{NH}}_{4}^{+}$ ha^−1^ as (NH_4_)_2_SO_4_ (δ^15^N = −0.1) to stimulate the growth and activity of soil nitrifiers. Plants were watered every second day (with 100 ml H_2_O), whereas the amount of water applied was doubled at 8, 19, and 28 days after N fertilization (DANF) in order to increase leaching of ${\text{NO}}_{3}^{-}$ derived from nitrification.

Rhizons (Soil Moisture Sampler, Ø 2.3 × 50 mm, porosity 0.1 μm, Eijkelkamp Agrisearch Equipment, Giesbeek, Netherlands) were installed horizontally into the pipes at 7.5 and 50 cm depth. This procedure enabled sampling of soil solution by suction pressure using a common medical syringe. Soil solution sampling (0, 3, 6, 7, 10, 14, and 20 DANF from topsoil and at 0, 18, and 27 DANF from 50 cm depth) was conducted at 3 h after irrigation with 100 ml of tap water to warrant sufficient soil moisture and time for establishing equilibrium for ${\text{NO}}_{3}^{-}$ level in the topsoil. A sample of 10 ml of soil solution was collected and frozen immediately. Concentration of ${\text{NO}}_{3}^{-}$ in soil solution samples was analyzed by complete reduction to ${\text{NO}}_{2}^{-}$ through hydrazine (in alkaline solution, with copper as catalyst) and subsequent reaction with sulfanilamide and Griess reagent (*N*-(1-*N*aphthyl)ethylenediamine) to form a pink compound measured photometrically at 550 nm (method DIN38405, ISO/DIS 13395; photometer: AutoAnalyzer 3, QuAAtro AQ2, SEAL Analytical Ltd., Southampton, United Kingdom).

Resin bags (ion-exchange resin in a fine nylon mesh) were installed at the bottom of the experimental pipe to trap leached ${\text{NO}}_{3}^{-}$. This allowed the quantification of cumulative ${\text{NO}}_{3}^{-}$-losses during the experiment. For this purpose, Resinex MX-11 (Jacobi Carbons GmbH, Frankfurt, Germany), a mixed anion/cation exchange resin with a maximum anion sorption capacity of 0.4 eq l^−1^ was used. Each bag contained 200 ml resin amended with 200 ml of washed sand in order to slow down percolate passage through the coarse resin matrix. The anion traps were removed at the end of the experiment (i.e., at 42 DANF). The resin was thoroughly homogenized and an aliquot of 40 ml resin of each bag was further processed. Extraction of ${\text{NO}}_{3}^{-}$ was performed twice with 200 ml of 2 M KCL and extracts were subsequently analyzed photometrically for ${\text{NO}}_{3}^{-}$ content (see analysis of percolate samples). A pre-test on the extractability of Resinex MX-11 verified that a twofold extraction was sufficient to achieve extraction rates of 98% of trapped ${\text{NO}}_{3}^{-}$.

At 42 DANF, soil solution sampling showed ${\text{NO}}_{3}^{-}$ levels below the detection limit using a fast ${\text{NO}}_{3}^{-}$ test (<5 mg ${\text{NO}}_{3}^{-}$ L^−1^). This indicated that the main effect of N fertilization should be reflected in terms of the δ^15^N and N uptake amount (mg N g plant dry matter^−1^). The aboveground shoot biomass (cut 3 cm above soil surface) of all seven Bh genotypes was sampled subsequently and oven-dried (3 days 60°C). Dry matter was determined and ground aliquots of plant material were filled into tin capsules (HEKAtech GmbH, Wegberg, Germany).

^15^N and N% were measured for all plant samples from Experiments 1–3 at UHOH by using a Euro Elemental analyzer coupled to a Finnigan Delta continuous-flow isotope ratio mass spectrometer IRMS (Thermo Scientific, Bremen, Germany). The ^15^N natural abundance of the sample relative to the standard (atmospheric N_2_) was expressed as: δ^15^N‰ = [(*R*_sample_/*R*_standard_) − 1] × 1000 (‰), where *R* represents the isotope ratio (^15^N/^14^N) and *R*_standard_ is ^15^N/^14^N for atmospheric N^2^ that is 0.0036765 (δ^15^N‰ = 0) (Robinson, 2001).

### Experiment 2: Differences in δ^15^N Leaf and Shoot of a Wide Range of Bh Genotypes Grown Under Field Conditions

A field trial was established in August 2013 with a range of Bh genotypes with contrasting BNI activity at Corpoica-La Libertad Research Center in the Piedmont region of Colombia (4°03′46” N, 73°27′47” W). The experimental field site was located at an altitude of 338 m above sea level with an annual mean temperature of 21.4°C and an average annual rainfall of 3,685 mm. The soil is classified as an Oxisol with a pH of 4.9, clay content of 42%, total N of 0.11%, and C/N ratio of 12.4. The trial included four out of the five Bh apomictic hybrids used in Experiment 1 and four Bh germplasm accessions. Each main plot had a size of 4 × 4 (16 m^2^) and received basal fertilization (kg ha^−1^) in September 2013 of 69 N (urea, δ^15^N = 0.05), 25 P, 50 K, 50 Ca, 15 Mg, 10 S, 0.5 B, and 2.6 Zn. The second N (100 kg N ha^−1^ as urea) fertilization (26 October 2015) was conducted after leaf sampling for Experiment 3 to stimulate both, BNI and soil nitrification.

For forage productivity and forage quality evaluation, grasses were cut every 6 weeks from October 2013 to November 2015. Aboveground shoot biomass samples of each plot were oven dried at 60°C. A sub-sample was ground and sent to UHOH for IRMS analysis (see above in Experiment 1).

### Experiment 3: δ^15^N of Young Regrown Bh Leaves Linked to BNI Indicated by Soil Incubation

Two years after initiation of the field trial described for Experiment 2, a substantial BNI effect was expected based on preliminary tests (soil incubation, data not shown). The grass was cut on 5 October 2015 (end of the rainy season) and all plots received maintenance fertilization (kg ha^−1^) of 40 P, 75 K, 110 Ca, 65 Mg, 19 S, 0.9 B, and 5.3 Zn. No N fertilizer was applied. It was intended that the plants take up soil mineral N and consequently reflect the δ^15^N of the soil mineral N. To avoid border effects during sampling, one sub-plot (1 m^2^) per main plot was randomly defined and marked. At 11 days after grass cutting (16 October), recently fully expended (second youngest) leaves from the regrown plants within the sub-plots were collected from the plots of three CIAT accessions as well as from Bh08 hybrids. Oven-dried and ground shoot samples were sent to UHOH for ^15^N and N% measurement (see above in Experiment 1).

Before cutting the grass in the field trial (Experiment 3) and adding the fertilizer in October 2015, topsoil samples (0–10 cm) were collected with an auger from eight randomly chosen points within each sub-plot. About 500 g of soil per sub-plot was thoroughly mixed, air-dried for 48 h, sieved (2 mm mesh size), and small stones as well as visible root material were removed. Representative sub-samples of 5 g of soil from each plot were filled in small glass tubes followed by application of 1.5 ml ammonium sulfate ((NH_4_)_2_SO_4_) solution as substrate for soil nitrifiers. Tubes were sealed with parafilm that contained two holes for aeration and placed in a dark incubation chamber with constant 25°C and 60% air humidity. Soil ${\text{NO}}_{3}^{-}$ was extracted before starting the incubation (basal) and after 5 and 25 days (main active phase of nitrification) with 50 ml of 1 M KCl. ${\text{NO}}_{3}^{-}$ concentrations were determined as described above (Experiment 1). NRs were expressed as mg N-${\text{NO}}_{3}^{-}$ kg dry soil^−1^ day^−1^.

### Statistical Analysis

Statistical analysis was performed using the SAS^®^ version 9.4 (SAS Institute Inc., Cary, NC, United States) with a mixed model approach. The assumptions of homogeneity of variance and normal distribution of residual errors were tested through the plots of studentized residuals vs. predicted value and quantile-quantile-plots, respectively, for all data sets. For the analysis of data from Experiment 2, the following model was set up: Genotype + Year + Rep + Genotype × Year. Block was set as random factor. Model-based least square means were used for data visualization with SigmaPlot for Windows version 12.0 (Copyright Systat Software, Inc.). The same software was also used for correlation analysis conducted for Experiments 1 and 3.

## Results

### Experiment 1 (Greenhouse at UHOH, Germany)

**FIGURE 1:**
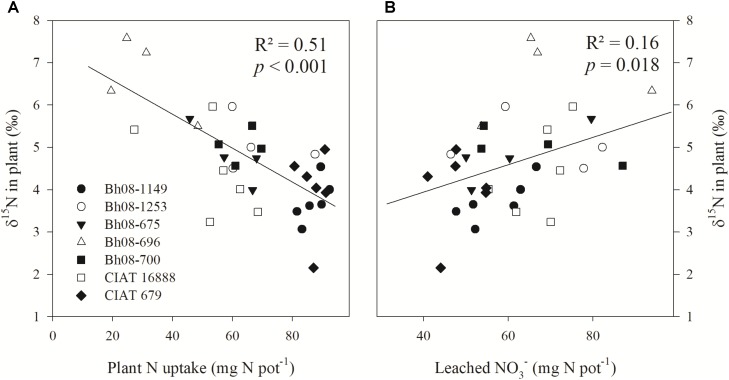
Linear regression of plant δ^15^N (‰) of *Brachiaria humidicola* (*Bh*) and plant N uptake (mg N pot^−1^) **(A)** and cumulative leached ${\text{NO}}_{3}^{-}$ (mg N pot^−1^) **(B)**. The greenhouse study (Experiment 1) included seven Bh genotypes and was established in August 2014. Plants were sampled at 42 days after fertilization (6 weeks after transplanting to experimental pots) with (${\text{NH}}_{4}^{+}$)_2_SO_4_ (δ^15^N = –0.1‰). Plant N uptake and leached ${\text{NO}}_{3}^{-}$ are cumulative amounts determined at the date of harvest.

Plant δ^15^N at harvest (42 DANF) was found to be negatively correlated with plant N uptake amounts (*p* < 0.001) (**Figure 1A**). Furthermore, the relationship between plant δ^15^N and the amount of cumulative leached ${\text{NO}}_{3}^{-}$ (*p* = 0.018; *R*^2^ = 0.16) was moderately positive (**Figure 1B**). In the case of low N uptake, plant δ^15^N was high, whereas plants with high N uptake were found to have lower plant δ^15^N (**Figure 1**). Increased leaching of ${\text{NO}}_{3}^{-}$-N lead to an increase of the δ^15^N of remaining N accumulating in the grass shoot biomass (**Table 1**). Furthermore, plant δ^15^N of all samples was relatively enriched compared to the δ^15^N of the applied ${\text{NH}}_{4}^{+}$ fertilizer (δ^15^N = −0.1). A linear regression analysis showed an inverse relationship (*p* < 0.001; *R*^2^ = 0.37) between plant N uptake and leached N (**Supplementary Material**).

**Table 1:** Plant δ^15^N (‰), plant N uptake (mg N pot^−1^), and leached N (mg N pot^−1^) at 42 days after N [(${\text{NH}}_{4}^{+}$)_2_SO_4_; δ^15^N = −0.1‰] fertilization of two *Brachiaria humidicola* (Bh) CIAT accessions and five Bh08 hybrids.

	CIAT 679	CIAT 16888	Bh08-675	Bh08-1149	Bh08-700	Bh08-696	Bh08-1253
Plant δ^15^N (‰)	4.08bc ± 0.34	4.35bc ± 0.34	4.72bc ± 0.42	3.80c ± 0.34	5.10b ± 0.42	6.70a ± 0.41	5.07b ± 0.42
Plant N uptake (mg N pot^−1^)	87.08a ± 1.62	53.52c ± 5.81	59.42bc ± 5.15	87.05a ± 1.73	63.16bc ± 3.16	30.94d ± 6.30	68.49b ± 6.54
Leached ${\text{NO}}_{3}^{-}$ (mg N pot^−1^)	48.27b ± 2.29	67.30a ± 3.00	60.30ab ± 6.83	57.07ab ± 3.07	66.08a ± 7.87	69.93a ± 8.52	66.43a ± 8.32
Dry matter (g)	7.13a ± 0.31	3.66c ± 0.39	4.19c ± 0.63	6.06ab ± 0.46	4.56bc ± 0.13	2.18d ± 0.24	4.23c ± 0.13

*Standard error of mean (±) is given for the mean values. Means that share a common letter within the same line do not differ significantly at the α = 5% level (LSD-test). Plants (six replications of CIAT 679 and CIAT 16888; four replications of Bh08 hybrids) were sampled at 42 days after N fertilization from a pot trial installed in a greenhouse at the UHOH (Experiment 1).*

### Experiment 2: Yearly δ^15^N Monitoring of Bh Genotypes in the Field (Field Trial La Libertad, Colombia)

**FIGURE 2:**
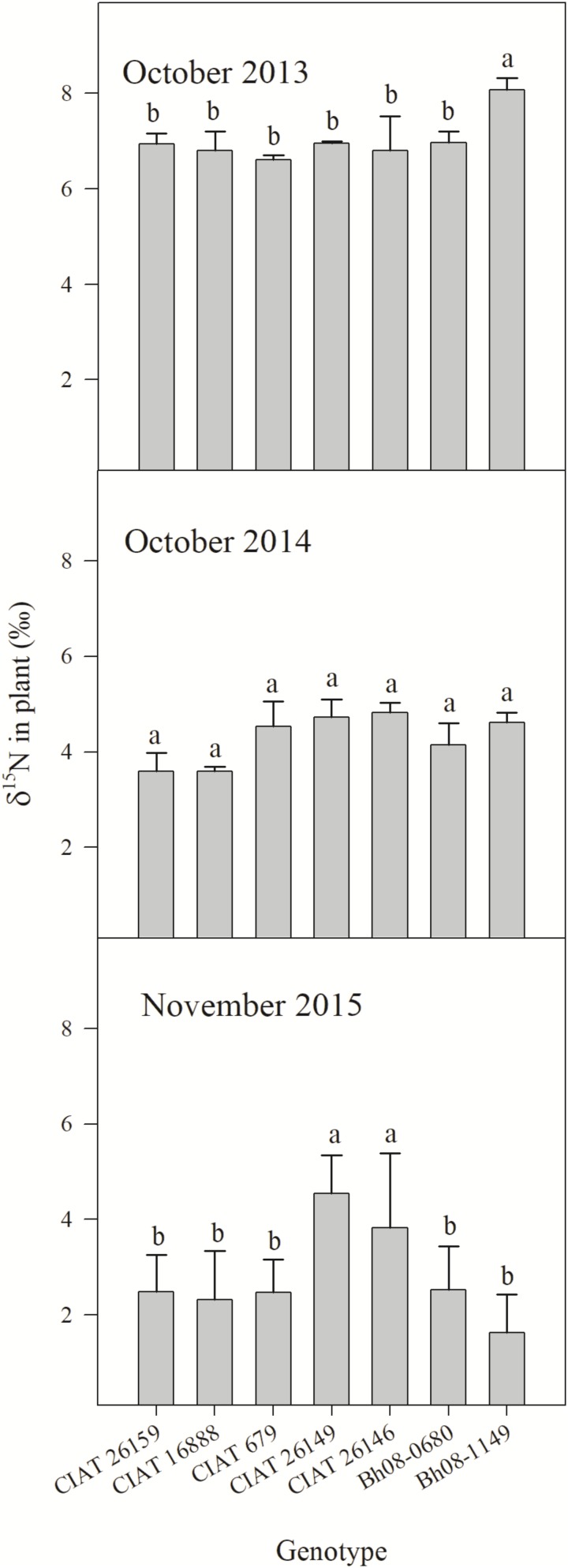
Plant δ^15^N (‰) of *B. humidicola* (*Bh*) of five contrasting (in terms of BNI) CIAT accessions and two hybrids (Bh08-population) with unknown BNI capacity sampled after the rainy season in the Colombian *Llanos* (Experiment 2). The field trail was established in August 2013. N fertilizer was applied as urea (δ^15^N = 0.05‰) in September 2013 (69 kg N ha^−1^) and in October 2015 (100 kg N ha^−1^). Error bars represent the standard error of the mean based on collected plants of three replicated completely randomized field plots. Means that share a common letter within the same year do not differ significantly at the a = 5% level.

The overall year effect showed an obvious trend of decreasing plant δ^15^N over the experimental seasons (*p* < 0.0001) (**Figure 2**). One month after experiment establishment and N fertilization (October 2013), most genotypes tended to a plant δ^15^N of around 7‰, except for the Bh08-1149 hybrid with a δ^15^N of almost 8‰. One year later (October 2014), δ^15^N of all genotypes had dropped below 5‰, however, a genotypic effect on δ^15^N abundance was absent (*p* = 0.13). At the last sampling (November 2015), δ^15^N of CIAT 26149 and CIAT 26146 had higher δ^15^N than the other three CIAT accessions and the two Bh08 hybrids (*p* = 0.02).

**FIGURE 3:**
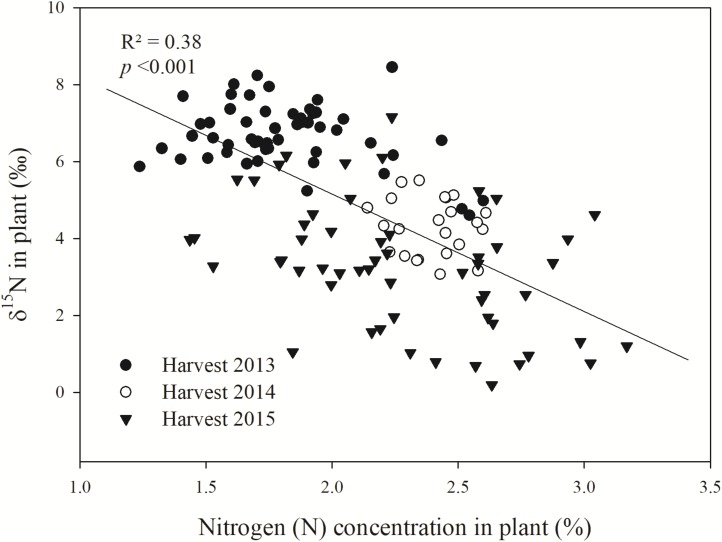
Linear regression of plant δ^15^N (‰) and plant N concentration (%) of contrasting *B. humidicola* (*Bh*) genotypes (in terms of BNI) sampled after the rainy season in the Colombian *Llanos* (Experiment 2). The field trail was established in August 2013. N fertilizer was applied as urea (δ^15^N = 0.05‰) in September 2013 (69 kg N ha^−1^) and in October 2015 (100 kg N ha^−1^).

To investigate an expected relationship between δ^15^N and N uptake by Bh, a regression analysis was conducted between the measured plant δ^15^N and the N concentration (N%) in the respective sampled Bh grass genotypes (**Figure 3**). A negative correlation (*p* < 0.001) was observed between plant δ^15^N and plant N%, indicating that the higher the N status of the plant is the lower the respective δ^15^N becomes.

### Experiment 3: Genotypic Leaf δ^15^N and Relation to Soil Nitrification (Field Trial La Libertad, Colombia)

**FIGURE 4:**
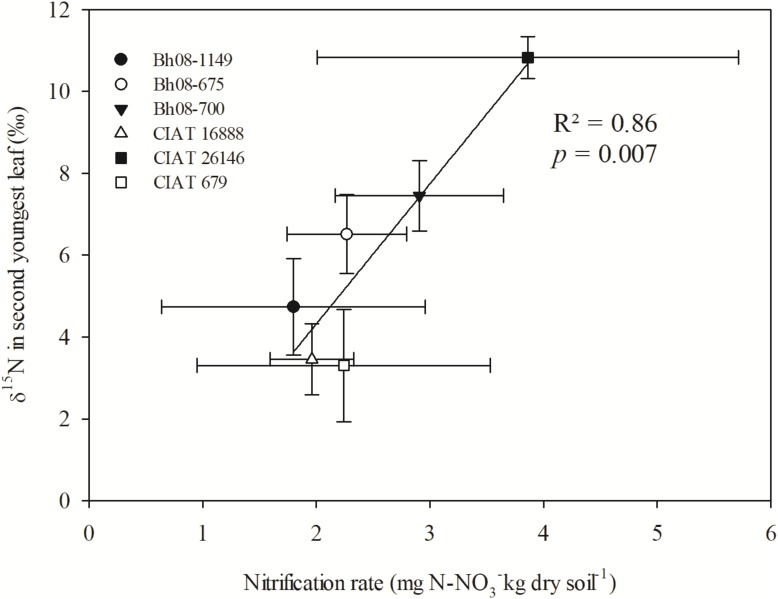
Linear regression of leaf δ^15^N (‰) of *B. humidicola* (*Bh*) and NRs (mg N-${\text{NO}}_{3}^{-}$ kg dry soil^−1^) determined from incubated soil sampled from the respective field plots (Experiment 3). The field trial included Bh CIAT accessions with potential of high BNI (CIAT 16888), mid-high BNI (CIAT 679), and low BNI (CIAT 26146). Additionally, three hybrids (Bh08-population) with unknown BNI capacity were sampled. The field trail was established in August 2013 in the Colombian *Llanos*. N fertilizer was applied as urea (δ^15^N = 0.05‰) in September 2013 (69 kg N ha^−1^). Plants were cut back at 5 October 2015 and topsoil (0–10 cm) samples were collected for incubation in the laboratory. Last fully developed leaves of Bh were sampled at 11 days after regrowth. Error bars represent the standard error of the mean based on collected leaf and soil samples of three replicated completely randomized field plots.

Means of δ^15^N values of regrown leaves at 11 days after regrowth (October 2015) of the selected Bh genotypes were positively correlated with the observed NRs of the respective Bh genotypes (*p* = 0.007) (**Figure 4**). Leaf δ^15^N means were found to be different among genotypes (*p* = 0.001), whereas the genotype effect was not significant for NRs (*p* = 0.74) (**Table 2**). In more detail, CIAT 26146 had highest δ^15^N leaf signal and the corresponding incubated soil showed highest NR. CIAT 16888 and CIAT 679, with reported high BNI, had, compared to CIAT 26146, significantly lower ^15^N leaf signals and were among the genotypes tested those with lower NRs.

**Table 2:** Nitrification rates (NRs) determined from incubated topsoil (0–20 cm, Oxisol, Colombian Llanos) sampled from field plots (October 2015) of three *B. humidicola* (Bh) CIAT accessions and three Bh hybrids (Experiment 3).

*B. humidicola* genotype	Nitrification rate mg N-${\text{NO}}_{3}^{-}$ kg soil day^−1^	SE	Plant δ^15^N (‰)	SE
CIAT 16888	2.24	1.3	3.3^a^	1.4
CIAT 679	1.96	0.4	3.5^a^	0.9
CIAT 26146	3.86	1.9	10.8^b^	0.5
Bh08-1149	1.80	1.2	4.7^a^	1.2
Bh08-700	2.91	0.7	7.5^ab^	0.9
Bh08-675	2.27	0.5	6.5^ab^	1.0

*Plant δ^15^N (‰) in regrown, second youngest leaves of the respective Bh plants (11 days after cutting and soil sampling for incubation). Genotype effect for NRs was not significant (p = 0.79). Mean values of three replications are shown (only two replications for plant δ^15^N of Bh08-675). Genotype effect for plant δ^15^N was significant (p = 0.02). Different letters indicate significant different means (α = 5%) according to multiple comparison procedures (Holm–Sidak method). SE, standard error of the mean.*

## Discussion

### Lower Plant δ^15^N Is Linked to Reduced ${\text{NO}}_{3}^{-}$ Losses and High Plant N Uptake

Our results of the greenhouse study confirmed the hypothesis that low plant δ^15^N is linked to enhanced N nutrition and reduced ${\text{NO}}_{3}^{-}$ leaching. Hence, the altered plant isotopic signals were indirectly linked to isotopic fractionation of ^15^N:^14^N between product (${\text{NO}}_{3}^{-}$) and substrate (${\text{NH}}_{4}^{+}$) during bacterial and archaeal nitrification (Delwiche and Steyn, 1970; Shearer et al., 1974). This led to subsequent leaching of the relatively ^15^N depleted ${\text{NO}}_{3}^{-}$. Our observations (Experiment 1) indicated that with increasing amounts of leached ${\text{NO}}_{3}^{-}$, the δ^15^N of the Bh grass increased (**Figure 1B**). These results suggest that losses of ^15^N depleted ${\text{NO}}_{3}^{-}$ resulted in relative enrichment of the plant available N. Similar observations of leaching processes leading to an ^15^N enrichment of the remaining soil ${\text{NH}}_{4}^{+}$ have been made by Pardo et al. (2007), Craine et al. (2009), and Stevenson et al. (2010). Furthermore, our observation of the positive relationship between increasing ^15^N:^14^N in vegetation due to increasing nitrification and N losses has also been described for leaves of forest trees (Pardo et al., 2002; Garten et al., 2008), cotton (Stamatiadis et al., 2006), mixed systems such as grass-heath-woodlands (Huber et al., 2013), as well as for comparative studies between perennial and annual grasses (Yé et al., 2015). Furthermore, evaluations at various sites demonstrated lower δ^15^N of ${\text{NO}}_{3}^{-}$ compared to δ^15^N of ${\text{NH}}_{4}^{+}$ in soil due to nitrification (Takebayashi et al., 2010). The yearly field evaluation revealed that ^15^N signals in Bh decreased with improved N status of the grass over time, indicating increased utilization of plant N as also observed in Central European grasslands by Kleinebecker et al. (2014). We suggest that in our field trial this was caused by decreasing ${\text{NO}}_{3}^{-}$ losses from the system due to expanded plant (e.g., root system) growth and development over the years.

### Long-Term BNI Effect in the Field Expressed in Low Plant δ^15^N

The general trend of decreasing plant δ^15^N of Bh genotypes in the field study over the years indicated that N isotope fractionation and consequently the δ^15^N of the mineral N in the soil changed over time. Thus, our results confirmed that during the early stage after establishment of the Bh genotypes, there was no significant influence of BNI on microbial nitrification. This observation is consistent with other studies on BNI expression with young Bh plants (Miranda et al., 1994; Castoldi et al., 2013; O’Sullivan et al., 2016). Therefore, it is suggested that applied urea N (hydrolyzed to ${\text{NH}}_{4}^{+}$ within a few days) during transplanting had a strong stimulation effect on growth and activity of soil nitrifiers, and that the microbial formed ${\text{NO}}_{3}^{-}$ was leached rapidly in the first rainy season when the grass was still small. BNI was unlikely to be strongly expressed, since plants were less than 2 months old and a strong BNI effect, due to an accumulation of BNI substances in the soil, needs about 1 year of Bh establishment (Nuñez et al., 2018). The high δ^15^N of Bh plants observed in October 2013 could therefore reflect the strong ^15^N enrichment of the soil mineral N pool caused by substantial nitrification and leaching loss of ^15^N depleted ${\text{NO}}_{3}^{-}$ during the establishment of the trial (Nadelhoffer and Fry, 1994; Song et al., 2014). Plant δ^15^N after the second rainy season (October 2014) were lower than the first sampling. BNI ability has been shown to be promoted with developing root biomass leading to less nitrification over time (Subbarao et al., 2009). Reduced N losses during the second rainy season explained the lower plant δ^15^N caused by a lower ^15^N enrichment of soil mineral N pool. The general tendency of decreasing ^15^N natural abundance of Bh genotypes over the years was also visible in the third year. This indicated further reduction of ${\text{NO}}_{3}^{-}$ formation and loss due to increase of BNI. This was verified by other soil incubation studies (Arango et al., unpublished data) revealing low NRs during the second (2014) rainy season. In contrast, incubation of soil sampled during the third rainy season (2015) evidenced significantly lower average NRs (3.5 mg N-${\text{NO}}_{3}^{-}$ kg dry soil^−1^ day^−1^). However, since root systems expand over the years a general higher uptake of ${\text{NH}}_{4}^{+}$ is expected. This could, additionally to BNI substance release, increase the competiveness of Bh for ${\text{NH}}_{4}^{+}$ and indirectly reduce nitrifier activity.

### Link Between High BNI and Low Leaf δ^15^N of Bh Genotypes

In our study, we linked leaf δ^15^N to BNI by Bh. It was evident that low nitrification in incubated soil taken from plots, where Bh was cultivated for more than 2 years, correlated with lower leaf δ^15^N. Exudation of brachialactone by Bh and other known nitrification inhibiting substances (Gopalakrishnan et al., 2009; Subbarao et al., 2009) are supposed to increase the relative ${\text{NH}}_{4}^{+}$-to-${\text{NO}}_{3}^{-}$ uptake, thereby reflecting primarily the δ^15^N signal of the plant available soil ${\text{NH}}_{4}^{+}$ pool (Kahmen et al., 2008). Furthermore, Bh root exudates have been shown to reduce *N. europaea* populations in soil (Gopalakrishnan et al., 2009). Our results thus support the hypothesis that high BNI (low NRs) results in lower leaf δ^15^N, as observed in our field study. Robinson (2001) suggested to measure whole plant δ^15^N when studies intend to indicate source δ^15^N in plant tissues to avoid uncertainties in isotopic discrimination during partitioning in the plant. In this respect, however, it has to be considered that intra-plant ^15^N discrimination (e.g., root-to-shoot) is generally small when N availability is low or when ${\text{NH}}_{4}^{+}$ is the primary mineral N form taken up by the plant (Evans, 2001).

### Different Plant δ^15^N Among Contrasting Bh Genotypes in Terms of BNI

Biological nitrification inhibition differences among Bh accessions or hybrids have been revealed (Subbarao et al., 2007; Rao et al., 2014; Nuñez et al., 2018) and the effect of high BNI is expected to reduce ${\text{NO}}_{3}^{-}$ formation and leaching (Subbarao et al., 2009, 2013). But experimental evidence for the latter is lacking. We observed a strong genotypic effect on leaf δ^15^N in our field studies. For instance, Bh genotypes with low NRs in the field (CIAT 16888, CIAT 679, CIAT 26159) showed a strong BNI effect from their root exudates on *N. europaea* (Subbarao et al., 2007). At the end of the field study, the same genotypes showed lower δ^15^N than CIAT 26149 with known low BNI potential (Subbarao et al., 2007). Furthermore, the higher leaf δ^15^N of CIAT 26146 compared to CIAT 16888 and CIAT 679 fit to our hypotheses, and earlier BNI evaluations (Subbarao et al., 2009; Nuñez et al., 2018). However, apart from BNI, other factors could have altered shoot δ^15^N of Bh genotypes. Exemplary is the acknowledged symbiosis of mycorrhizae with plants (Evans, 2001). However, under low N availability the cycling of N through the fungus to the plant is rather negligible for plant δ^15^N (Högberg et al., 1999).

## Conclusion

We studied the interlinkages of plant δ^15^N, BNI, microbial nitrification, N uptake, and N leaching losses under controlled as well as under field conditions based on a selection of contrasting Bh genotypes. Our main conclusion is that high BNI activity decreases plant δ^15^N of Bh. Thus, the ^15^N natural abundance of grass tissue might be linked to BNI activity in soil, suppressing the growth and activity of bacterial and archaeal nitrifiers. As a result, this led to enhanced ${\text{NH}}_{4}^{+}$ uptake by Bh and reduced ${\text{NO}}_{3}^{-}$ losses. This ecological concept is enhanced if: (i) BNI is expressed in soil due to long-term presence of Bh; (ii) there is a continuous substrate (${\text{NH}}_{4}^{+}$) supply (mineralization, fertilization) so that the source N for plant uptake is never converted completely (into ${\text{NO}}_{3}^{-}$); and (iii) a significant amount of ${\text{NO}}_{3}^{-}$ formed by nitrification is leached of the rooting zone of the plants. Since Bh can take up both N forms (Castilla and Jackson, 1991), the cumulative ^15^N shoot signal would be confounded in case of ${\text{NH}}_{4}^{+}$ and ${\text{NO}}_{3}^{-}$ uptake without nitrate loss. Furthermore, other microbial enzymatic reactions should not mask the discrimination process by nitrifiers, such as: (i) volatilization (driven by high soil pH, heat, not incorporated N fertilizer); (ii) denitrification (anaerobic conditions, high C availability, ${\text{NO}}_{3}^{-}$ substrate present). However, nitrate substrate left would even be higher enriched in ^15^N; and (iii) uptake of ^15^N depleted N derived by free living N fixing bacteria.

Our observations suggest that high BNI along with reduced microbial nitrification (one of the main reactions causing ^15^N:^14^N fractionation) and N leaching (enriching the remaining soil mineral N with ^15^N) are reflected in low δ^15^N leaf or shoot biomass signals under environments with high ${\text{NO}}_{3}^{-}$ leaching potential. We also suggest that the method described here can serve as an indicator of the extent of ${\text{NO}}_{3}^{-}$ leakiness for BNI field evaluations over the years, if combined with other BNI indicators like the abundance and activity of soil nitrifiers under the given conditions set out above.

## Author Contributions

HK wrote the manuscript and had the overall task to modify it according to suggestions and corrections of the co-authors. Furthermore, HK measured all samples with the IRMS that have been used for this study. KE installed the greenhouse trial (Experiment 1) and conducted the sampling. JN assisted in sampling of plant material (Experiment 3) and delivered further plant material from other experiments for pre-tests (data not shown). IR was the leading senior scientist of the BMZ project at CIAT Colombia. FR contributed to the scientific interpretation and the concept of the study. JA was the leading young professional of the BMZ project at CIAT Colombia and enabled HK the access to the field trial of Experiments 2 and 3 for sampling. DM had the responsibility of taking samples of Experiment 2. AA assisted in the incubation study of Experiment 3. GC was the leading senior scientist of this study. All authors contributed to manuscript revision, read, and approved the submitted version.

## Conflict of Interest Statement

The authors declare that the research was conducted in the absence of any commercial or financial relationships that could be construed as a potential conflict of interest.
