# Supplementary material for: Low 15N Natural Abundance in Shoot Tissue of Brachiaria humidicola Is an Indicator of Reduced N Losses Due to Biological Nitrification Inhibition (BNI)
Source: Front Microbiol. 2018 Oct 4;9:2383. doi: 10.3389/fmicb.2018.02383 (PMC6186998; doi:10.3389/fmicb.2018.02383)
Supplement: Supplementary file 1 [file Data_Sheet_1.PDF]

## *Supplementary Material*

# Low $^{15}N$ Natural Abundance in Shoot Tissue of *Brachiaria humidicola* is an Indicator of Reduced N Losses due to Biological Nitrification Inhibition (BNI)

**Hannes Karwat, Konrad Egenolf, Jonathan Nuñez, Idupulapati Rao, Frank Rasche, Jacobo Arango, Danilo Moreta, Ashly Arevalo, Georg Cadisch***

*** Correspondence:** Georg Cadisch: georg.cadisch@uni-hohenheim.de

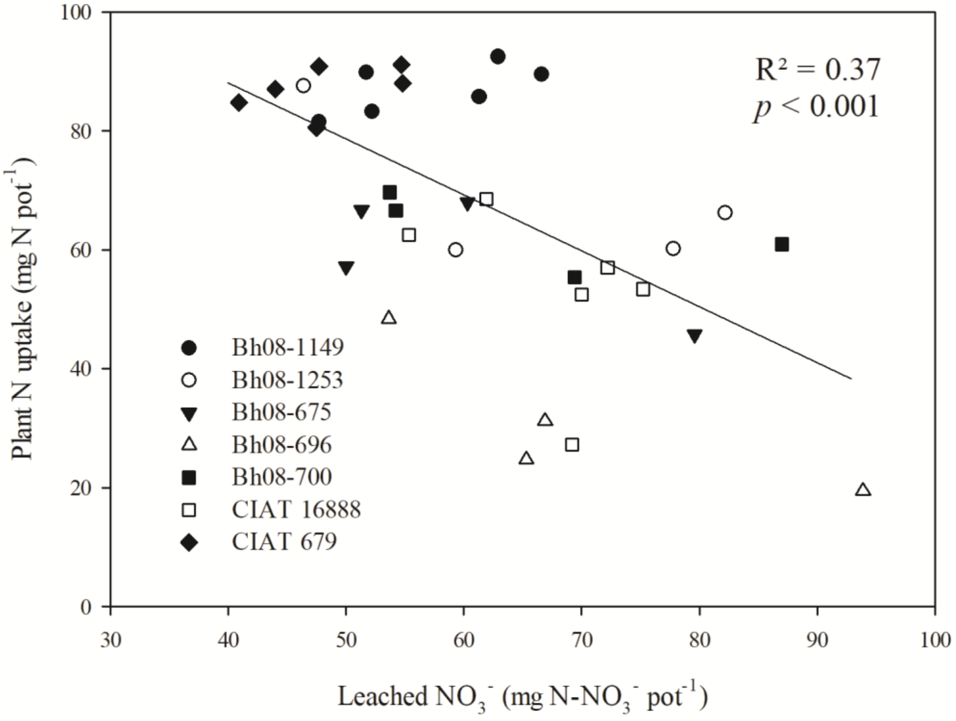


**Supplementary Figure 1.** Linear regression of plant N uptake (mg N $pot^{-1}$) of *Brachiaria humidicola* (*Bh*) and cumulative leached $NO_3^-$ (mg N $pot^{-1}$). The greenhouse study (Experiment 1) included 7 *Bh* genotypes and was established in August 2014. Plants were sampled at 42 days after fertilization (6 weeks after transplanting to experimental pots) with $(NH_4^+)_2SO_4$. Plant N uptake and leached $NO_3^-$ are cumulative amounts determined at the date of harvest.
